# The stabilization potential of a standing molecule

**DOI:** 10.1126/sciadv.abj9751

**Published:** 2021-11-10

**Authors:** Marvin Knol, Hadi H. Arefi, Daniel Corken, James Gardner, F. Stefan Tautz, Reinhard J. Maurer, Christian Wagner

**Affiliations:** 1 Peter Grünberg Institut (PGI-3), Forschungszentrum Jülich, 52425 Jülich, Germany.; 2 Jülich Aachen Research Alliance (JARA)–Fundamentals of Future Information Technology, 52425 Jülich, Germany.; 3 Experimentalphysik IV A, RWTH Aachen University, Otto-Blumenthal-Straße, 52074 Aachen, Germany.; 4 Department of Chemistry, University of Warwick, Gibbet Hill Road, CV4 7AL Coventry, UK.

## Abstract

The part-by-part assembly of functional nanoscale machinery is a central goal of nanotechnology. With the recent fabrication of an isolated standing molecule with a scanning probe microscope, the third dimension perpendicular to the surface will soon become accessible to molecule-based construction. Beyond the flatlands of the surface, a wealth of structures and functionalities is waiting for exploration, but issues of stability are becoming more critical. Here, we combine scanning probe experiments with ab initio potential energy calculations to investigate the thermal stability of a prototypical standing molecule. We reveal its generic stabilization mechanism, a fine balance between covalent and van der Waals interactions including the latter’s long-range screening by many-body effects, and find a remarkable agreement between measured and calculated stabilizing potentials. Beyond their relevance for the design and construction of three-dimensional molecular devices at surfaces, our results also indicate that standing molecules may serve as tunable mechanical gigahertz oscillators.

## Teaser

Competing chemical and van der Waals interactions keep a molecule in the vertical until it is knocked over by heat.

## INTRODUCTION

**Fig. 1.**
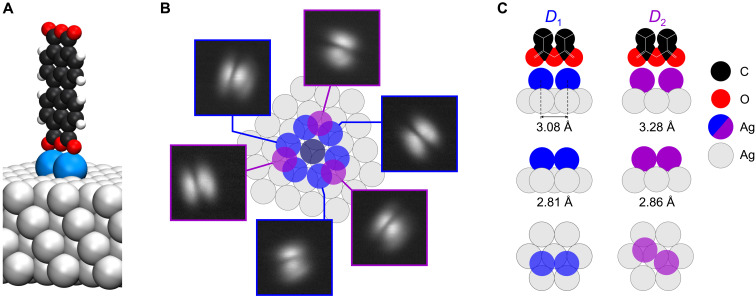
Geometry of the metastable *s*-PTCDA configuration. (**A**) Three-dimensional model of the standing PTCDA molecule (black, carbon atoms; red, oxygen atoms; white, hydrogen atoms) on two Ag adatoms (blue) on the Ag(111) surface (gray). (**B**) STM images (−50 mV, 0.2 nA, 25 × 25 Å^2^) of all six observable azimuthal orientations of *s*-PTCDA, linked to the respective adatom dimers, *D*_1_ (blue) or *D*_2_ (purple). The center adatom (gray) is part of all dimers. Three *D*_2_ dimers and six *D*_1_ dimers with pairwise identical azimuthal orientations can form in this way. (**C**) Top and side views of the two observed adatom pedestals, *D*_1_ and *D*_2_, with adatom distances computed by PBE + vdW^surf^ for the bare dimer (center row) and the dimer with PTCDA attached (top row).

The scanning probe microscope (SPM) has brought the vision of molecular-scale fabrication closer to reality, because it offers the capability to rearrange atoms and molecules on surfaces, thereby allowing the creation of metastable structures that do not form spontaneously (*1*–*7*). It was recently demonstrated that a single 3,4,9,10-perylene-tetracarboxylic dianhydride (PTCDA) molecule can be brought into a standing configuration on a Ag(111) surface (*4*) (Fig. 1A), if a pedestal of two adatoms is first created: Once the adatoms have been attached to one side of the flat-lying molecule by atomic manipulation, the SPM tip contacts a carboxylic oxygen atom (O_carb_) at the opposite side and lifts PTCDA on a curved trajectory into the vertical (see Materials and Methods). Two functionalities of this metastable SPM-fabricated device that are intricately related to the standing configuration have already been identified: Standing PTCDA (from now on referred to as *s*-PTCDA) acts as a single electron field emitter on the Ag(111) surface (*4*), and it functions as a quantum dot when attached to the Ag-covered SPM tip, thus enabling a new microscopy technique—scanning quantum dot microscopy (SQDM) (*8*–*10*).

While vertical molecular alignments in certain types of self-assembled monolayers (*11*–*13*) have been studied in detail, the stabilization mechanism of an isolated standing molecule, such as PTCDA on either the surface (*4*) or the tip (*8*–*10*), is not yet known. However, a detailed understanding of the stability of this prototypical structure is essential, both for the purposeful design of similar devices, in particular with the help of virtual design methodologies, and for the integration of SPM-based fabrication approaches with other nanofabrication methods. We therefore measure here the thermal stability of *s*-PTCDA up to a temperature of *T* = 14 K and, from these data, determine the potential energy barrier that stabilizes the molecule in its standing configuration. Complementary density functional theory (DFT) calculations reveal that this barrier results from a fine balance between covalent and van der Waals (vdW) interactions. Because the experimentally measured barriers are in the range of only 30 meV, *s*-PTCDA incidentally also serves as a highly sensitive benchmarking system for state-of-the-art ab initio theory.

## RESULTS

In scanning tunneling microscopy (STM), *s*-PTCDA appears as two separated elliptical features (Fig. 1B). These represent a side view of its lowest unoccupied molecular orbital, which features electron density at the O_carb_ atoms in the four corners of the PTCDA molecule and a nodal plane that is oriented perpendicular to the molecule and runs along its long axis. Six different azimuthal orientations of *s*-PTCDA have been observed (Fig. 1B) (*4*). They can be associated with two nonequivalent configurations, *D*_1_ and *D*_2_, of the adatom pedestal and their rotational equivalents (Fig. 1C).

**Fig. 2.**
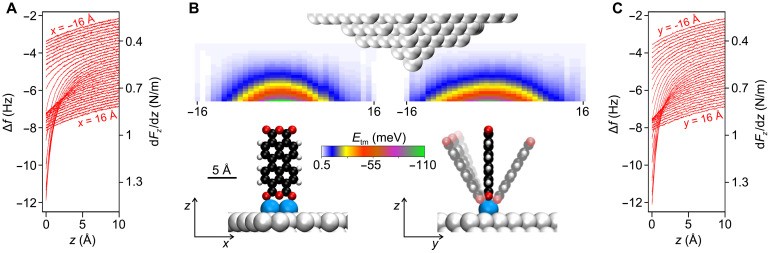
Tip-molecule interaction potential. (**A**) The 34 Δ*f*(*z*) curves measured at positions (−16 Å < *x* < 16 Å, *y* = 0) along a line centered above *s*-PTCDA at (*x* = 0, *y* = 0) and oriented along the PTCDA plane. Each curve is averaged over 10 individual spectra and offset vertically for better visibility (0.14 Hz between consecutive curves). (**B**) Sections of the tip-molecule interaction energy *E*_tm_ (*x*, *y*, *z*) along the molecular plane (left) and perpendicular to it (right) as obtained from the data in (A) and (C). Maps and *s*-PTCDA models are drawn and located to scale. (**C**) The 34 Δ*f*(*z*) curves similar to (A) but measured at positions (*x* = 0, −16 Å < *y* < 16 Å) on a line oriented perpendicular to the PTCDA plane.

It was found before that attempts to topple over the standing molecule with the SPM tip were unsuccessful (*4*). The data in Fig. 2, recorded at the base temperature *T* = 5 K of our instrument, show that this observation can be explained by an attraction between the tip and the standing molecule that persists long after the chemical tip-O_carb_ bond is broken. Sections of this long-range interaction energy *E*_tm_(*x*, *y*, *z*) between tip and molecule are displayed in Fig. 2B. Note that the molecular models that are depicted below the data are true to scale in size and position. The maps were obtained by twice integrating the d*F_z_*/d*z*(*z*) curves in Fig. 2 (A and C), recorded with a qPlus (*14*) noncontact atomic force microscope (AFM; see Materials and Methods). The data show that in the *y* direction, the *E*_tm_ profile is broader. This has been assigned to a bending displacement of the molecule in response to the attraction of the tip (*4*), as shown schematically below the corresponding map. Because there is no visible discontinuity in the *E*_tm_(*y*, *z*) map, we conclude that *s*-PTCDA either returns smoothly to the vertical when the tip is retracted or snaps back unnoticed, that is, at *E*_tm_ well below 10 meV. This points to a very shallow stabilizing potential of the standing molecule.

Given this finding of a rather weak stabilization, we can expect to obtain the potential depth from lifetime measurements of *s*-PTCDA in a temperature range that is actually achievable in SPM experiments without compromising long-term stability. To cope with the observed tip-molecule attraction (Fig. 2), we have conceived a measurement protocol based on repeated tip retractions. While the attraction by the tip allows erecting *s*-PTCDA even at temperatures at which its intrinsic lifetime is vanishingly small, the tip retraction eliminates any interactions and allows observing the intrinsic stability of *s*-PTCDA.

**Fig. 3.**
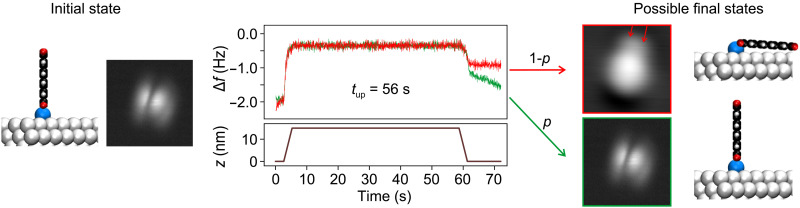
Measurement protocol for the probability of *s*-PTCDA collapse. Exemplary measurement protocol for the probability *p* of *s*-PTCDA to remain vertical over a time *t*_up_. The SPM tip, initially stabilizing the molecule, is retracted within 3 s by 15 nm, parked for 53 s, and then re-approached (brown curve). The time *t*_up_ = 56 s is defined as the time at which *z* ≥ 7.5 nm. The Δ*f* signal after re-approach indicates whether the molecule collapsed (red) or remained vertical (green) while the tip was retracted. The nonzero Δ*f*(*t*) slope at the end of the green curve results from piezo-creep in the presence of a strong Δ*f*(*z*) gradient (Fig. 2A). STM imaging (25 × 25 Å^2^) confirms the state of the molecule. Because the adatom dimer is not affected by the collapse, the orientation of the toppled molecule (red box, location of Ag dimer marked by red arrows) reveals the orientation of *s*-PTCDA just before the collapse.

The aim of the lifetime measurements is to determine the probability *p*(*t*, *T*) that molecules remain standing for a time *t* at temperature *T* after the tip has been retracted. Instead of analyzing an ensemble of molecules, we use a single molecule that we erect repeatedly. The measurement protocol is shown in Fig. 3. After erecting the molecule and breaking the bond between tip and O_carb_, we keep the tip close to the molecule such that the attractive tip-molecule interaction *E*_tm_ stabilizes *s*-PTCDA. Then, we rapidly retract the tip by Δ*z* = 15 nm, which switches off its stabilizing influence on the standing molecule. After *t* = *t*_up_, the tip is brought back to its initial position. Depending on the frequency shift signal Δ*f*, we determine whether the standing molecule has survived the time period *t*_up_ or collapsed. Repeating the experiment many times, we determine *p*(*t*_up_, *T*) from the number *s* of survivals divided by the number *n* of trials at the given temperature *T*.

**Fig. 4.**
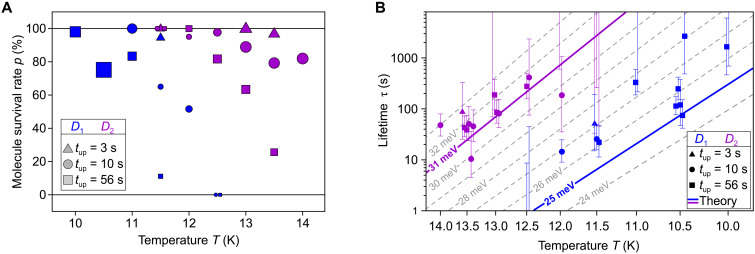
Temperature-dependent stability of *s*-PTCDA. (**A**) Survival rate *p* = *s*/*n* of *s*-PTCDA molecules on *D*_1_ (blue) and *D*_2_ (purple) pedestals as a function of sample temperature *T* and duration of tip retraction *t*_up_. The symbol size (area) is proportional to the number of trials *n* on which the respective data point is based (min = 10, max = 200). Higher survival rates facilitate more trials because PTCDA has to be re-erected less frequently. (**B**) Arrhenius plot of the temperature-dependent lifetimes τ of *D*_1_- and *D*_2_-type *s*-PTCDA obtained with Eq. 1 for each individual set of experiments. Sets of trials in which the molecule either always or never dropped yield apparent lifetimes of τ = 0 or τ = ∞ such that the corresponding data points are outside the plot. The uncertainty in τ is quantified by the 95% Wilson confidence intervals or by the rule of three (*31*) in cases where *t* = 0 or *t* = ∞ (see Materials and Methods). Data points at the same temperature are laterally offset for better visibility. The gray isolines represent τ (*T*) relations for a set of barrier heights 24 meV ≤ *E*_A_ ≤ 33 meV (Eq. 2) including the barrier heights obtained by DFT for *D*_1_ (blue) and *D*_2_ (purple). For the isolines, the attempt frequency $\omega_{0}(T)=2.06\cdot 10^{9}s^{-1}K^{-\frac{1}{2}}\cdot T^{\frac{1}{2}}$ was used.

In total, we have detected 235 collapses while performing a total of 1270 trials at nine different temperatures in the interval 10 K ≤ *T* ≤ 14 K and three different *t*_up_ times (3, 10, and 56 s). The *p*(*t*_up_, *T*) data are summarized in Fig. 4A. The plot shows a dependence of *p* not only on *T* and *t*_up_ but also on the type of *s*-PTCDA. Molecules on a *D*_2_ pedestal survive temperatures that are about 2 K higher than molecules on *D*_1_ do. A 60:40 occurrence ratio of *D*_1_ and *D*_2_ molecules was observed previously in unbiased manipulation experiments (*4*), whereas a 2:1 ratio would be expected if both orientations had the same potential energy landscape, because the absolute number of hollow-site combinations for *D*_1_ pedestals is twice as large as for *D*_2_ (Fig. 1B). We can thus conclude that *s*-PTCDA with a *D*_2_ pedestal is not only more likely to be created but also thermally more stable.

In our experiments, we find a complete correlation between the azimuthal orientations of *s*-PTCDA and the toppled molecule (see the STM images in Fig. 3). This correlation proves that *s*-PTCDA always collapses by rotating around the adatom-adatom axis of the pedestal. Furthermore, it also allows us to clear the statistics of cases in which a *D*_2_ molecule switches spontaneously to *D*_1_ and then collapses immediately due to its lowered thermal stability (see Materials and Methods).

The statistical data in Fig. 4A can be converted into lifetimes τ of the standing molecule. To this end, we assume first-order kinetics d*N*/d*t* = − *kN* of the transition *s*-PTCDA → PTCDA, where *N* is the number of standing molecules. Identifying *N*(*t*, *T*)/*N*_0_ with *p*(*t*_up_, *T*) = *s*(*t*_up_, *T*)/*n*, this yields

$$\tau (T)=k^{-1}(T)=-\frac{t_{\text{up}}}{\text{ln}\;(p(t_{\text{up}},T))}$$ (1)

The result is shown in the Arrhenius plot of Fig. 4B. Each data point corresponds to a set of trials, recorded for one combination of *t*_up_ and *T* in a given run, such that there can be multiple sets for the same combination (*t*_up_, *T*). Within their uncertainty intervals, the data for *D*_1_ and *D*_2_ largely obey the linear trend expected for a thermally activated reaction with

$$\tau^{-1}=\omega_{0}e^{-E_{A}/k_{B}T}$$ (2)

With an attempt frequency ω_0_ obtained from transition state theory (see below), the average activation energies *E*_A_ range between ∼26 meV for *D*_1_ molecules and ∼31 meV for *D*_2_ molecules. These tiny barriers indicate an extremely shallow stabilization potential of the standing molecule. Ab initio calculations, to which we now turn, quantitatively confirm the measured barriers and in particular the difference between *D*_1_ and *D*_2_ molecules.

In the standing configuration, the center of mass of PTCDA resides at a distance of approximately 7 Å from the surface plane of the substrate. Hence, the overlap between the electronic densities of the molecule and the metal is small, allowing for example the mentioned applications of *s*-PTCDA in SQDM and as a field emitter. With respect to the electronic structure calculations, the weakness of the coupling increases the relevancy of long-range vdW forces for a proper description of the system. At the same time, the anticipated shallowness of the potential requires the highest possible accuracy. In our dispersion-inclusive DFT calculations using the Perdew-Burke-Ernzerhof (PBE) functional (*15*), we therefore compare two state-of-the-art approaches to describe the vdW interaction, namely, the vdW^surf^ (*16*) and many-body dispersion (MBD) (*17*, *18*) schemes. In combination with PBE, both are thoroughly benchmarked and widely used to describe molecule-metal hybrid systems (*19*–*22*). In the vdW^surf^approach, one sums over density-dependent pairwise interactions (*23*) that include the effective screening by the metallic substrate up to the lowest-order two-body dispersion energy (*16*). In MBD, on the other hand, the screened long-range many-body vdW energy is calculated beyond the pairwise approximation by representing the long-range polarization response of the system as a set of dipole-coupled quantum harmonic oscillators (*17*, *18*). To ensure internal consistency, we perform all structural relaxations separately for vdW^surf^ and MBD.

The adatoms of *D*_1_ pedestals occupy two adjacent hollow sites of the same type [face-centered cubic (fcc) or hexagonal close-packed (hcp) sites, respectively] (*4*) with a hollow-site distance *d*_1_ = 2.89 Å, while *D*_2_ pedestals occupy nonidentical hollow sites (fcc-hcp combination) with a distance *d*_2_ = 3.34 Å (Fig. 1C). We first compare the relaxed geometries (PBE + vdW^surf^) of the two dimer types, both with and without *s*-PTCDA on top (Fig. 1C). Without *s*-PTCDA, the relaxed *D*_1_ and *D*_2_ dimers have adatom separations *d*_1_ = 2.81 Å and *d*_2_ = 2.86 Å, both of which are smaller than the distances of the respective pairs of hollow sites, indicating a substantial adatom-adatom attraction. When *s*-PTCDA is attached on top, Ag-O_carb_ bonds form and the adatom distances increase to *d*_1_ = 3.08 Å and *d*_2_ = 3.28 Å. This indicates that the Ag-O_carb_ bonds decouple the adatoms from each other to some extent, suggesting a substantial directionality of the former. This provides the first hint toward a possible stabilization mechanism.

**Fig. 5.**
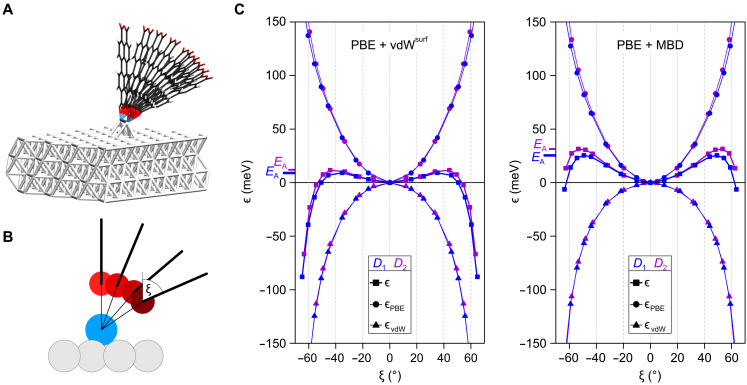
Potential energy profile of *s*-PTCDA. (**A**) Set of molecular configurations used in the *s*-PTCDA configuration-space mapping. (**B**) View along the dimer axis. Constrained geometry optimization at every tilt angle ξ reveals that PTCDA rotates neither strictly around the Ag adatoms nor around the O_carb_ atoms. The (polar) angle of the Ag-O_carb_ bond is slightly smaller than the angle ξ of the molecular plane (thick black line). The bond lengths are not drawn to scale. (**C**) Relative total energy curves ϵ(ξ) = *E*(ξ) − *E*(0 ° ) for the PBE + vdW^surf^ (left) and PBE + MBD method (right). The contributions to ϵ = ϵ_PBE_ + ϵ_vdW_ are plotted individually. The barrier height *E*_A_, which is the maximal ϵ value obtained for each case, is indicated.

The calculations confirm the experimental finding (Fig. 2) that the primary degree of freedom of *s*-PTCDA is an essentially rigid rotation around the adatom-adatom axis (tilt angle ξ). We thus calculate the potential energy profiles along ξ (Fig. 5A) and account for the small translations required to optimize the geometry of the Ag-O_carb_ bonds at each tilt angle ξ by constrained geometry optimization (Fig. 5B). The subtle difference between *D*_1_ and *D*_2_ mandates calculations with very tight convergence criteria (see Materials and Methods). The resulting potential energy curves ϵ(ξ) = *E*(ξ) − *E*(0^∘^) in Fig. 5C show a qualitatively similar behavior for both configurations, *D*_1_ and *D*_2_, and for both vdW methods. They reveal a shallow minimum at the vertical alignment of *s*-PTCDA and a steep decrease of the potential energy for absolute values ∣ξ∣ beyond 40° to 50^∘^. Both regions are separated by a barrier *E*_A_, which ranges from 9 meV for *D*_1_ using vdW^surf^ to 31 meV for *D*_2_ and MBD. Separating ϵ = ϵ_PBE_ + ϵ_vdW_ into PBE and vdW contributions (Fig. 5C), we find a pronounced minimum in ϵ_PBE_(ξ) at ξ = 0^∘^. This points to a substantial directionality of the local bonding situation that is captured by the PBE calculation. In contrast, the nonlocal long-range molecule-surface attraction ϵ_vdW_(ξ) draws the molecule into the horizontal.

With the help of transition state theory (*24*, *25*), we determine the lifetime τ of *s*-PTCDA in the calculated potential wells, treating it as a rigid rotor with moment of inertia *I* (Materials and Methods). Although the differences between the potentials in Fig. 5C are small in absolute terms, the corresponding lifetimes differ by orders of magnitude. For example, at the experimental temperature *T* = 5 K, the *D*_1_ molecule has a vdW^surf^-calculated lifetime of only 0.1 s, while MBD predicts τ = 1.7 × 10^15^ s. We thus conclude that only the potentials calculated with MBD are consistent with the experimental observation of a standing molecule at 5 K.

## DISCUSSION

It has been shown that calculations with a combination of PBE and MBD can provide accurate predictions of the stability of molecule-metal interfaces (*19*) with the typical range of accuracy for cohesive energies at δ*E* ∼ 45 meV (approximately 1 kcal/mol) (*26*). At first sight, it is unexpected that the activation energy *E*_A_ can be predicted with an agreement of δ*E* ∼ 1 meV (Fig. 4B), and such a level of accuracy can certainly not be generalized to a wide class of problems. Moreover, a fortuitous cancellation of systematic errors in ϵ and *T* of a few millielectron volts cannot be excluded. However, the activation energies are given as differences ϵ_PBE_ − ∣ϵ_vdW_∣ between energy differences ϵ(∣ξ_b_∣) = *E*(∣ξ_b_∣) − *E*(0), i.e., *E*_PBE_(∣ξ_b_∣) − *E*_PBE_(0) − ∣ *E*_vdW_(∣ξ_b_∣) − *E*_vdW_(0)∣, where ∣ξ_b_∣ is the tilt angle at which the barrier reaches its maximum. While the four energies in the above expression may each have an absolute systematic error of the order δ*E*, the two differences *E*_PBE_(∣ξ_b_∣) − *E*_PBE_(0) and ∣*E*_vdW_(∣ξ_b_∣) − *E*_vdW_(0)∣ are both more accurate than δ*E* and also relatively small. As a consequence, their difference evidently reaches a level of predictive accuracy that permits the differentiation not only between the state-of-the-art methods to calculate the vdW energy but also between the bonding situations on the *D*_1_ and *D*_2_ pedestals. Regarding the latter, Fig. 5C not unexpectedly shows that the difference primarily stems from the local bonding as calculated by PBE. The more stretched-out *D*_2_ pedestal provides stiffer bonds.

Regarding the comparison between vdW^surf^ and MBD, we note that the difference in the absolute surface energies in combination with PBE is only 12% or less in the investigated ξ range, in agreement with calculations for flat-lying PTCDA (*19*). This deviation, however, causes a 170% increase in the predicted barrier heights (9 and 12 meV for *D*_1_ and *D*_2_, respectively, with vdW^surf^ versus 25 and 31 meV with MBD), with drastic consequences for the lifetime. Thus, *s*-PTCDA provides an extremely sensitive benchmarking system for the calculation of vdW interactions. Specifically, the benchmark reveals that the possibility to erect the standing molecule at *T* = 5 K is a direct consequence of the screening of the long-range vdW interaction by nonadditive many-body effects (*17*, *18*). This screening reduces the attraction to the surface and ultimately stabilizes the molecule in the upright geometry because the higher-order contributions to the vdW energy contained in MBD counteract the pairwise contributions that are present in vdW^surf^. Because the former decay faster with distance, the screening becomes stronger as the molecule tilts.

Last, the experimentally validated potentials ϵ(ξ) show that *s*-PTCDA is a mechanical oscillator with an estimated frequency of 23 GHz for *D*_1_ and 26 GHz for *D*_2_. These values result when using the calculated moment of inertia *I* and scaling the calculated ϵ(ξ) in Fig. 5C to the experimentally estimated barrier heights of 26 and 31.5 meV, respectively. We expect that with a (harmonic oscillator) level spacing of Δ*E* = *hf* = 0.1 meV, this oscillation mode is constantly excited even at our base temperature of 5 K. Because the tip-molecule interaction potential (Fig. 2B) further deepens the potential, the tip can be used to continuously tune the resonance frequency of *s*-PTCDA up over a range of several tens of gigahertz.

In conclusion, we have measured the lifetime of a three-dimensional SPM-fabricated single-molecule device and identified its stability mechanism as a generic balance between local covalent interactions and nonlocal long-range vdW forces. The results reported here may guide the future design and fabrication of a broad range of metastable molecular structures. The remarkable correspondence between calculated and measured stabilization potentials proves that ab initio calculations with chemical accuracy will be able to support this design process. The predicted mechanical oscillation mode of *s*-PTCDA is located in the gigahertz energy range where also magnetic excitations are typically investigated (*27*). This is of particular interest, because *s*-PTCDA has also been shown to host an unpaired local spin (*28*, *29*). We anticipate that *s*-PTCDA, which is a 1-nm-sized gateable quantum dot and tunable gigahertz oscillator, may yet become a fascinating component in the toolbox of quantum nanoscience.

## MATERIALS AND METHODS

### Experiments

Experiments were carried out in a commercial UHV LT-STM/AFM system (Createc) at a base temperature of 5 K using a third-generation qPlus sensor (*14*) with *k* = 1800 N/m, *f*_0_ = 31.2 kHz, and *Q* = 46,000. The tip is a PtIr wire of 25 μm diameter and 130 μm length cut with a focused ion beam.

The fabrication of *s*-PTCDA proceeded as described in (*4*): (i) By standard atomic manipulation with the SPM tip, two Ag adatoms were moved sequentially into the vicinity of the two adjacent O_carb_ atoms on one of the short edges of an isolated flat-lying PTCDA molecule to which they bind spontaneously. (ii) The SPM tip was brought in contact with one of the O_carb_ atoms on the opposite side of the molecule. (iii) By virtue of the spontaneously formed tip-molecule bond, the molecule was lifted into the vertical using an appropriately curved tip trajectory. (iv) Once the molecule reached the vertical, the tip was moved straight up. This broke the tip-molecule bond and left the molecule standing on the adatom pedestal (Fig. 1A). After a collapse, the Ag_2_ PTCDA complex remains intact and can be brought back into the vertical orientation by molecular manipulation as described above (steps ii to iv).

To obtain the tip-molecule potential in Fig. 2B, Δ*f*(*z*) spectra for (*x*, *y*) positions along two lines (*x* = −19…+ 19 Å and *y* = 0) and (*x* = 0 and *y* = −19…+ 19 Å) were measured. For both lines, 10 Δ*f* (*z*) spectra at each of 40 locations along the line (Fig. 2, A and C) were recorded, starting at *z* = *z*_0_ and moving away from the surface with a vertical tip speed of 2 Å/s. Each batch of 10 Δ*f* (*z*) spectra at one position was averaged to minimize low-frequency noise. To eliminate the contribution of the attraction between the tip and the sample surface, the first and last three spectra (i.e., the six spectra recorded furthest away from *s*-PTCDA) were averaged to obtain a reference that was subtracted from the remaining 34 Δ*f* (*z*) spectra. The tip-molecule force gradients were calculated as d*F_z_*/d*z*(*z*) = − 2*k*/*f*_0_Δ*f* (*z*), with *k* = 1800 N/m and *f*_0_ = 31.2 kHz. The resulting d*F_z_*/d*z*(*z*) curves were integrated twice to obtain *E*_tm_(*x*, *y*, *z*) in two planes as shown in Fig. 2B. The absolute tip height of *z*_0_ ≈ 5 Å above *s*-PTCDA (models in Fig. 2B) was estimated from tip approach curves in which contact with *s*-PTCDA was established. The attractive tip-molecule potential shown in Fig. 2B stabilizes *s*-PTCDA at elevated temperature as long as the tip is not retracted, i.e., also during SPM imaging at sufficiently small image size (15 × 15 Å^2^, as done regularly to check the state of the molecule).

We observed occasional spontaneous switches of the *s*-PTCDA configuration between *D*_1_ and *D*_2_. Therefore, the orientation of *s*-PTCDA was verified by STM imaging after every five tip retraction cycles and after each collapse of *s*-PTCDA. We found a ∼5% probability of *s*-PTCDA changing its orientation between two consecutive orientation checks. It is also possible to switch *s*-PTCDA deliberately between *D*_1_ and *D*_2_ by positioning the tip at one side of the molecule and applying a voltage of −0.35 V for several seconds until the molecule switches in a way that places one O_carb_ atom right beneath the tip.

The tip-molecule interaction cannot be switched off instantaneously by tip retraction. Because it decays gradually with tip height (Fig. 2), it is instead smoothly reduced to zero as the tip is retracted by Δ*z* = 15 nm. The time *t*_up_, which affects the calculated lifetimes via Eq. 1, is thus not uniquely defined. To approximately account for the time with nonvanishing tip-molecule interaction, we sum 50% of the retraction and re-approach periods into *t*_up_. We have taken several further measures to mitigate the effect of finite retraction and approach speeds: (i) The potential in Fig. 2 decays to a few millielectron volts at a tip height of approximately 2 nm above the molecule. Yet, we retract the tip by 15 nm, because very weak but very long ranged tip-molecule interactions might be present that would be inaccessible to the measurements of Fig. 2. (ii) We retract at high tip speeds of 5 to 7 nm/s, thus traversing the 2-nm range of nonvanishing tip-molecule interactions within 0.3 to 0.4 s. This is short even compared to the smallest *t*_up_ value of 3 s. (iii) We performed experiments for three very different *t*_up_ times, namely, 3, 10, and 56 s, and found no related systematic deviations in the data in Fig. 4B. This confirms the effectiveness of our approach.

An uncertainty in τ arises from the finite number *n* of trials in each set. For sets with 0 < *s* < *n*, this uncertainty was quantified by the 95% Wilson confidence interval for binomial distributions according to which the upper and lower bounds of the Wilson confidence interval are given as τ_±_ = − *t*_up_/ ln (*p*_±_) with

$$p_{\pm }=\frac{s+c^{2}/2}{n+c^{2}}\pm \frac{z}{n+c^{2}}\sqrt{\frac{s(n-s)}{n}+\frac{c^{2}}{4}}$$ (3)

where *c* = 1.96 for the 95% confidence interval (*30*). For sets with *s* = 0 or *s* = *n* where the computed lifetime is either zero or infinite, the 95% confidence interval was calculated by the “rule of three,” which states that in a binomial distribution the lower bound of the 95% confidence interval for the probability *p* of an event that was always observed in *n* trials is given by *p* = 1 − 3/*n*, while the upper bound of the 95% confidence interval for the probability *p* of an event that was never observed in *n* trials is given by *p* = 3/*n* (*31*). The error bars in Fig. 4B depict the 95% confidence intervals.

### DFT calculations

DFT calculations were performed with the all-electron numeric-atomic-orbital–based “Fritz-Haber-Institute ab initio molecular simulation package (FHI-aims)” (*32*). The semi-local exchange-correlation functional PBE (*15*) was used to treat the electronic exchange and correlation. To account for the dispersion effects, we used both, the vdW^surf^ scheme (*16*, *23*) and MBD (*17*, *18*), during geometry optimization and for single-point energy calculations. To include relativistic effects, the scaled zeroth-order regular approximation (ZORA) (*33*) was applied in all calculations.

The Ag(111) surface was modeled by a four-layer 8 × 8 silver slab consisting of 256 Ag atoms. A 60 Å vacuum layer was inserted between repeated slabs stacked along the *z* direction. Starting from the experimental surface lattice constant (*a* = *b* = 2.89 Å), the lattice parameter was first converged for a four-layer Ag(111) primitive unit cell, and the supercell was constructed accordingly. The converged lattice parameter (*a* = *b* = 2.875 Å) is very close to the experimental value. For *D*_1_ and *D*_2_, the two silver adatoms were initially placed at the respective hollow sites, 2.36 Å above the surface and *d*_1_ = 2.89 Å and *d*_2_ = 3.34 Å apart, respectively. We defined the reaction coordinate ξ for the rotation around the axis of adatoms as the angle between the plane of PTCDA and the surface normal. The bottom three layers of the slab were held fixed, while the top layer, the adatoms, and PTCDA were allowed to relax freely except for the constraint discussed below.

In all calculations, periodic boundary conditions were applied with a Γ-centered 2 × 2 × 1 Monkhorst-Pack grid (*34*) to sample the Brillouin zone during structural relaxation. It was replaced with a denser 4 × 4 × 1 mesh for DFT + vdW^surf^/MBD single-point calculations. For the MBD postprocessing calculations, the Brillouin zone was sampled with a half-Γ–shifted 8 × 8 × 1 *K*-points grid. To aid the convergence, a 0.02 eV broadening was applied to all states, using a Gaussian occupation smearing scheme.

All structures were relaxed with the Broyden-Fletcher-Goldfarb-Shanno (BFGS) algorithm in two steps. First, the Kohn-Sham wave function was expanded with the default numerical “light” basis sets (*35*) applied to all atomic species and the structures were relaxed. Once finished, all basis sets were replaced with the default numerical “tight” (*35*) basis sets and the relaxation was resumed. The relaxation was continued until the maximum force on each atom, in either setup, was less than 10^−3^ eV/Å. To obtain a well-converged electronic description, a threshold of 10^−7^ eV for the total energy, 10^−4^ eV for the sum of eigenvalues, and 10^−6^ e/Å^3^ for the charge density was applied during all self-consistent field cycles.

When mapping the potential ϵ(ξ), a structural relaxation was carried out while keeping the tilt angle ξ of the molecule approximately fixed. This constraint was imposed by fixing the *z* coordinate of a single carbon atom at the edge of the PTCDA molecule far from the Ag-O_carb_ bond (its *x* and *y* coordinates were left free). Because the relaxation of the Ag-O_carb_ bond under this constraint may lead to a small change in ξ, the value of ξ was remeasured for the relaxed structure, and this value was used in the plots in Fig. 5C.

### Transition state theory calculations

The standing molecule was treated as a rigid rotor with moment of inertia *I*. The attempt frequency in Eq. 2 then becomes $\omega_{0}={\left(k_{B}T/2\pi I\right)}^{\frac{1}{2}}$. The moment of inertia *I* was calculated as $I=\sum_{i}m_{i}r_{i}^{2}=31082\;g\;\text{mo}l^{-1}{Å}^{2}$. The barrier heights *E*_A_ in Eq. 2 were obtained by cubic spline interpolation of the DFT data in Fig. 5C. The two pathways of escape from the potential well (i.e., toppling over to the left or right in Fig. 2B) were accounted by summing over the contributions of the individual pathways, which due to the symmetry of the potential well leads to a twofold increase in the rate calculated for a single pathway.
